# Study on the Therapeutic Effects and Mechanisms of Gintonin in Irritable Bowel Syndrome and Its Relationship with TRPV1, TRPV4, and NaV1.5

**DOI:** 10.3390/ph17091170

**Published:** 2024-09-04

**Authors:** Na-Ri Choi, Seok-Jae Ko, Joo-Hyun Nam, Woo-Gyun Choi, Jong-Hwan Lee, Seung-Yeol Nah, Jae-Woo Park, Byung-Joo Kim

**Affiliations:** 1 Department of Longevity and Biofunctional Medicine, Pusan National University School of Korean Medicine, Yangsan 50612, Republic of Korea; nariring@gmail.com (N.-R.C.); ak0510@hanmail.net (W.-G.C.); 2 Department of Korean Medical Science, Pusan National University School of Korean Medicine, Yangsan 50612, Republic of Korea; 3 Department of Clinical Korean Medicine, Graduate School of Kyung Hee University, Seoul 02447, Republic of Korea; kokokoko119@daum.net; 4 Department of Gastroenterology, College of Korean Medicine, Kyung Hee University, Seoul 02447, Republic of Korea; 5 Department of Physiology, Dongguk University College of Medicine, Kyungju 38066, Republic of Korea; ferinus@gmail.com; 6 Channelopathy Research Center (CRC), Dongguk University College of Medicine, Goyang 10326, Republic of Korea; 7 Department of Biomedical Engineering, Dong-Eui University College of Engineering, Busan 47340, Republic of Korea; jonghwanlee@deu.ac.kr; 8 Ginsentology Research Laboratory and Department of Physiology, College of Veterinary Medicine, Konkuk University, Seoul 05029, Republic of Korea; synah@konkuk.ac.kr

**Keywords:** gintonin, irritable bowel syndrome, zymosan, gastrointestinal disease, ion channels

## Abstract

Irritable bowel syndrome (IBS) is a gastrointestinal (GI) disease accompanied by changes in bowel habits without any specific cause. Gintonin is a newly isolated glycoprotein from ginseng that is a lysophosphatidic acid (LPA) receptor ligand. To investigate the efficacy and mechanisms of action of gintonin in IBS, we developed a zymosan-induced IBS murine model. In addition, electrophysiological experiments were conducted to confirm the relevance of various ion channels. In mice, gintonin restored colon length and weight to normal and decreased stool scores, whilst food intake remained constant. Colon mucosal thickness and inflammation-related tumor necrosis factor-α levels were decreased by gintonin, along with a reduction in pain-related behaviors. In addition, the fecal microbiota from gintonin-treated mice had relatively more *Lactobacillaceae* and *Lachnospiraceae* and less *Bacteroidaceae* than microbiota from the control mice. Moreover, gintonin inhibited transient receptor potential vanilloid (TRPV) 1 and TRPV4 associated with visceral hypersensitivity and voltage-gated Na^+^ 1.5 channels associated with GI function. These results suggest that gintonin may be one of the effective components in the treatment of IBS.

## 1. Introduction

Irritable bowel syndrome (IBS) is a gastrointestinal (GI) disease accompanied by changes in bowel habits, such as abdominal pain, abdominal distension, diarrhea, and constipation, that are repeated without any specific cause [1]. No obvious structural or biochemical abnormalities have been reported to account for these symptoms [2,3]. Various epidemiological studies demonstrate that women and teenagers are susceptible to IBS [4]. In comparison to other chronic diseases such as diabetes mellitus, IBS reduces the quality of life [2]. The prevalence of IBS varies greatly from country to country. According to a 2017 Rome Foundation announcement, the prevalence of IBS varies greatly from country to country [5]. The degree of IBS can be divided into mild (1–36 points), moderate (37–110 points), and severe (≥111 points) according to the validated functional bowl severity disorder index (FBSDI) [6]. IBS can be subclassified into IBS-C, with main symptoms of constipation, pain, and discomfort; IBS-D, whose predominant symptom is diarrhea; IBS-M, with a mix of constipation and diarrhea; and IBS-U, which is unsubtyped [7]. Currently, IBS treatment involves changes in dietary habits and lifestyle. There are also medications that change depending on the symptoms the patient complains of. In addition, psychiatric treatment includes cognitive–behavioral therapy, relaxation therapy, and hypnosis. However, there is still no definitive treatment method [8]. Moreover, the incidence of IBS was reported to increase after Coronavirus disease 2019 [9]. Therefore, increasing our understanding of IBS is important to choose better treatment for patients with IBS, as well as to reduce medical costs.

Ginseng has been well-used for centuries as a tonic in traditional medicine [10,11]. Recently, gintonin, a lysophosphatidic acid (LPA) receptor ligand, was extracted from ginseng and a glycoprotein that has various physiological and pharmacological effects [11]. Among the many effects, gintonin is known to have a good effect on Alzheimer’s diseases by oral administration [12,13]. It is also said that gintonin is also effective in the GI tract. Gintonin depolarizes pacemaker potentials of interstitial cells of Cajal (ICC) through LPA1/LPA3 receptor signaling pathways in small intestine [14]. In addition, gintonin has a protective effect against gastric ulcers [15]. Despite evidence that gintonin has various functions in the GI tract, studies on IBS have not yet been conducted.

In this study, we used zymosan to create a murine model of IBS with symptoms similar to those of human IBS and verified that gintonin exhibited protective effects on the colon, suppressed inflammation, and modulated the fecal microbiome. In addition, gintonin regulated various ion channels related to the visceral hypersensitivity of IBS. Therefore, this study suggests the possibility of treating IBS-like hypersensitivity with gintonin, a healthy ginseng ingredient with fewer side effects, although it has no definite definition as a treatment method for IBS.

## 2. Results

### 2.1. Subsection Effects of Gintonin on Colon and Stool Changes in the Zymosan-Inducd Murine Model

**Figure 1:**
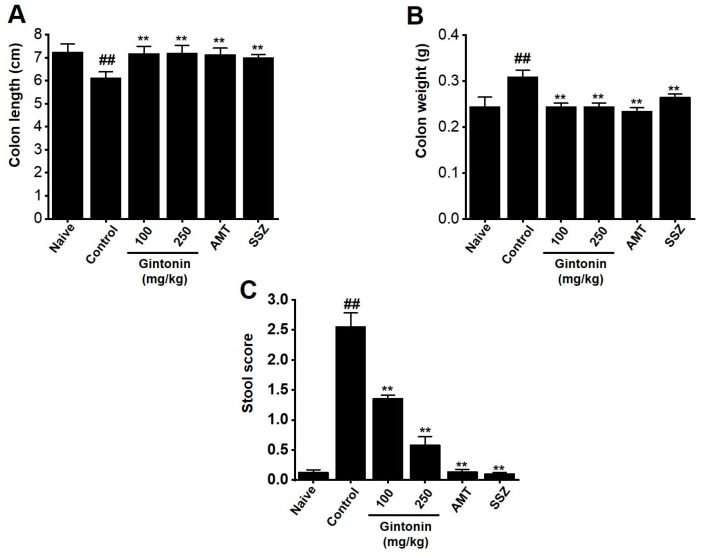
Effects of gintonin on the colon in mice with zymosan-induced IBS. (**A**) Colon length. (**B**) Colon weight. (**C**) Stool score. Bars represent the mean ± standard error of the mean. ## *p* < 0.01 vs. naïve. ** *p* < 0.01 vs. control.

We characterized changes in the colon and stool. Amitriptyline (AMT), an antidepressant drug, and sulfasalazine (SSZ), an anti-inflammatory drug, were used as positive controls for this experiment [16,17]. Zymosan administration reduced colon length (Figure 1A). However, the colon length following gintonin treatment was restored to that of the naïve (non-IBS) mice (naïve 7.25 ± 0.34 cm, control 6.13 ± 0.25 cm (*p* < 0.01), gintonin (100 mg/kg) 7.19 ± 0.32 cm (*p* < 0.01), gintonin (250 mg/kg) 7.21 ± 0.33 cm (*p* < 0.01), AMT 7.15 ± 0.27 cm (*p* < 0.01), and SSZ 7.00 ± 0.14 cm (*p* < 0.01); Figure 1A). In addition, in the zymosan control group, colon weight was increased compared to that in the naïve group. However, gintonin restored colon weight in line with the non-IBS mice (naïve 0.25 ± 0.02 g, control 0.31 ± 0.01 g (*p* < 0.01), gintonin (100 mg/kg) 0.24 ± 0.01 g (*p* < 0.01), gintonin (250 mg/kg) 0.25 ± 0.02 g (*p* < 0.01), AMT 0.24 ± 0.01 g (*p* < 0.01), and SSZ 0.27 ± 0.01 g (*p* < 0.01), Figure 1B). The stools of naïve mice exhibited a firm, brown consistency, whereas zymosan-induced mice had light brown or yellow watery diarrhea, resulting in high stool scores. Treatment with gintonin restored the stool score to levels similar to those of non-IBS mice (naïve 0.13 ± 0.04, control 2.55 ± 0.24 (*p* < 0.01), gintonin (100 mg/kg) 1.36 ± 0.06 (*p* < 0.01), gintonin (250 mg/kg) 0.58 ± 0.14 (*p* < 0.01), AMT 0.14 ± 0.04 (*p* < 0.01), and SSZ 0.10 ± 0.02 g (*p* < 0.01); Figure 1C). These results suggest that gintonin reverses zymosan-induced colitis and diarrhea.

### 2.2. Effects of Gintonin on Body Weight and Food Intake Changes in the Zymosan-Induced Murine Model

**Figure 2:**
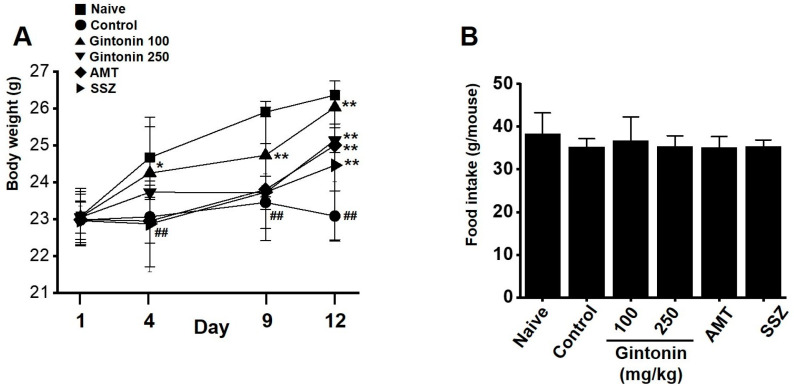
Effects of gintonin on body weight and food intake in mice with zymosan-induced IBS. (**A**) Body weight. (**B**) Food intake. Bars represent the mean ± standard error of the mean. ## *p* < 0.01 vs. naïve. * *p* < 0.05, ** *p* < 0.01 vs. control.

Control mice exhibited weight loss compared to naïve mice on days 4, 9, and 12. However, if gintonin was administered, zymosan-treated mice had weights similar to those of naïve mice on day 4 (naïve 24.67 ± 1.09 g, control 23.06 ± 0.71 g (*p* < 0.01), gintonin (100 mg/kg) 24.25 ± 1.25 g (*p* < 0.05), gintonin (250 mg/kg) 23.73 ± 0.19 g, AMT 22.95 ± 1.39 g, and SSZ 22.87 ± 1.16 g; Figure 2A), day 9 (naïve 25.90 ± 0.13 g, control 23.45 ± 0.71 g (*p* < 0.01), gintonin (100 mg/kg) 24.73 ± 1.46 g (*p* < 0.01), gintonin (250 mg/kg) 23.72 ± 0.12 g, AMT 23.73 ± 1.32 g, and SSZ 23.80 ± 0.42 g; Figure 2A), and day 12 (naïve 26.36 ± 0.38 g, control 23.08 ± 0.68 g (*p* < 0.01), gintonin (100 mg/kg) 26.03 ± 0.45 g (*p* < 0.01), gintonin (250 mg/kg) 25.14 ± 0.34 g (*p* < 0.01), AMT 24.46 ± 2.02 g (*p* < 0.01), and SSZ 25.00 ± 0.99 g (*p* < 0.01); Figure 2A). However, no difference in food intake was observed between the treatments over 12 days (Figure 2B). These results suggest that gintonin prevented weight loss in the zymosan-induced IBS mice.

### 2.3. Effects of Gintonin on Zymosan-Induced Colitis

**Figure 3:**
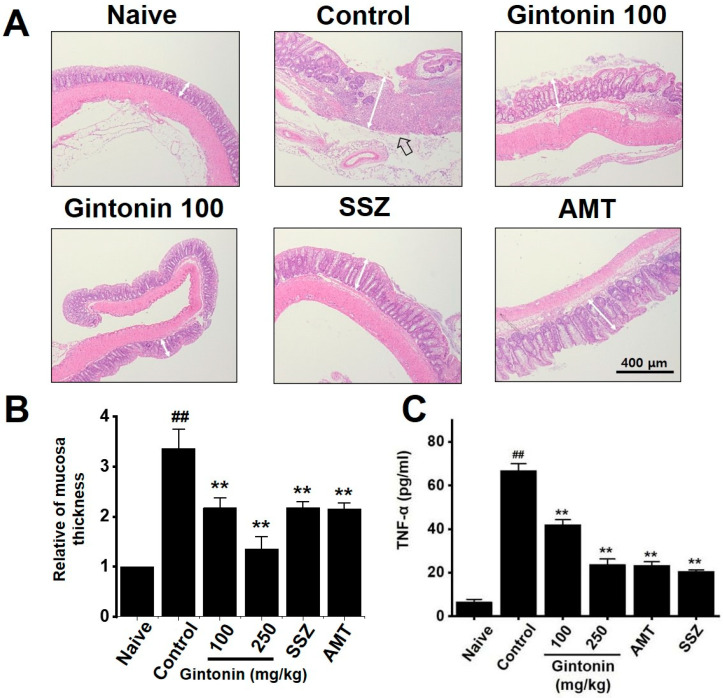
Effects of gintonin on mucosa and tumor necrosis factor (TNF)-α levels in mice with zymosan-induced IBS. (**A**) Hematoxylin and eosin staining. (**B**) Relative mucosal thickness. (**C**) TNF-α levels. Bars represent the mean ± standard error of the mean. ## *p* < 0.01 vs. naïve. ** *p* < 0.01 vs. control. Black arrow: inflammatory cell infiltration. White bars: epidermal thicknesses.

Past studies have demonstrated that zymosan causes inflammation of the colon, as indicated by mucosal thickening [18,19]. In this study, H&E staining confirmed an increase in the thickness of the colon mucosa and inflammatory cell infiltration after zymosan administration, indicative of inflammation and IBS. This increase in colon mucosal thickness was negated by gintonin administration (Figure 3A,B). In addition, TNF-α levels, which were increased in zymosan-induced IBS, were reduced by gintonin (Figure 3C). These results suggest that gintonin suppressed colonic inflammation in the zymosan-induced mice with IBS.

### 2.4. Effects of Gintonin on Pain-Related Behaviors

**Figure 4:**
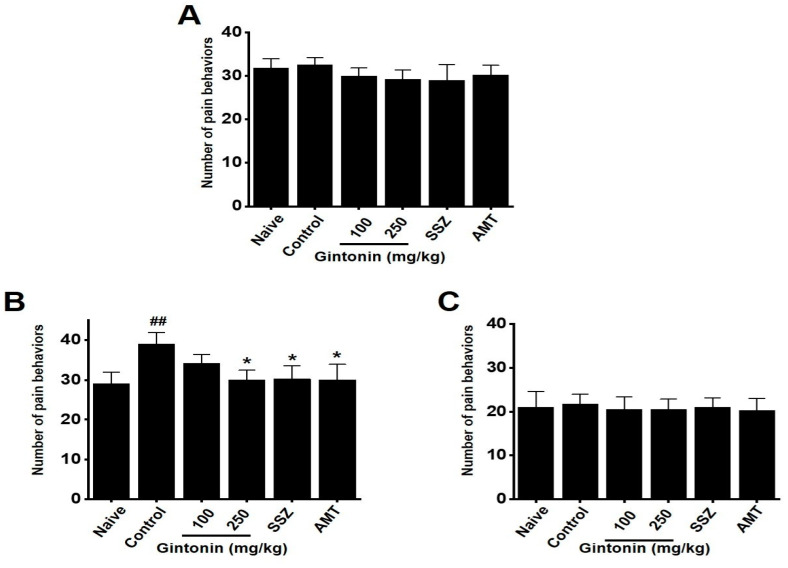
Effects of gintonin on pain-related behaviors in mice with zymosan-induced IBS. The number of pain-related behaviors was measured at (**A**) 1, (**B**) 9, and (**C**) 12 days. Bars represent the mean ± standard error of the mean. ## *p* < 0.01 vs. naïve. * *p* < 0.05 vs. control.

On the first day, pain-related behaviors did not appear (Figure 4A). However, by day 4, the zymosan-treated mice began to exhibit pain-related behaviors. These behaviors were reduced by day 9 in mice administered gintonin (naïve 29.11 ± 2.94, control 39.12 ± 2.95 (*p* < 0.01), gintonin (100 mg/kg) 34.25 ± 2.20, gintonin (250 mg/kg) 30.13 ± 2.45 (*p* < 0.05), AMT 30.25 ± 3.30 (*p* < 0.05), and SSZ 30.15 ± 3.91 (*p* < 0.05), Figure 4B). The pain-related behaviors disappeared by day 12 (Figure 4C). These results suggest that gintonin alleviated pain in zymosan-induced mice with IBS.

### 2.5. Effect of Gintonin on the Composition of Fecal Microorganisms

**Figure 5:**
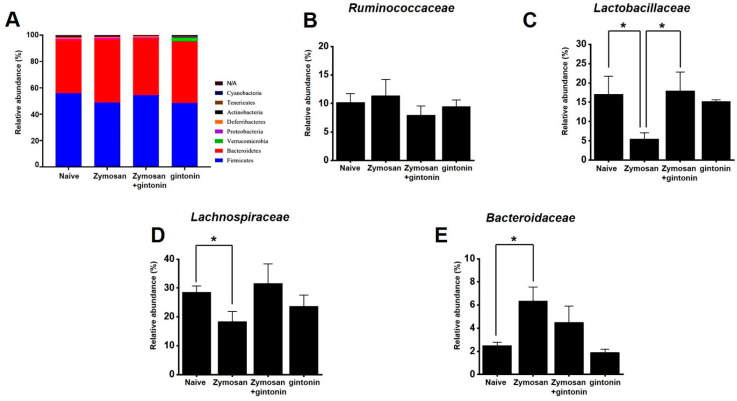
Relative abundance of fecal microbiota phyla and families. (**A**) Bar graph representing the relative bacterial phylum abundance. Relative abundance of (**B**) *Ruminococcaceae*, (**C**) *Lactobacillaceae*, (**D**) *Lachnospiraceae*, and (**E**) *Bacteroidaceae* families in each group. * *p* < 0.05.

To determine whether gintonin affected the composition of the fecal microbiome, the feces of each experimental group were analyzed. Phylum-level changes were observed in microorganisms between the groups (Figure 5A). At the family level, no specific change was identified in *Ruminococcaceae* (Figure 5B). However, gintonin increased *Lactobacillaceae* (Figure 5C) and *Lachnospiraceae* (Figure 5D), although the increase was not significant. Additionally, *Bacteroidaceae* were reduced, but not significantly (Figure 5E).

### 2.6. Effects of Gintonin on TRPV1, TRPV4, and TRPA1 Channel Currents

**Figure 6:**
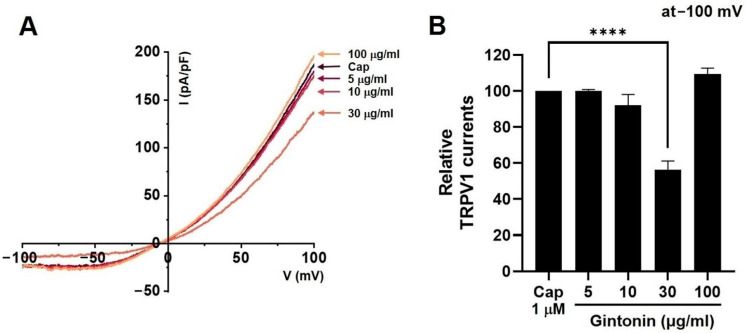
Effects of gintonin on overexpressed transient receptor potential vanilloid (TRPV)1 channel currents in HEK293T cells. (**A**) Representative I-V curve displaying the effects of various gintonin concentrations on overexpressed TRPV1 currents (I_TRPV1_). (**B**) Statistical analysis of relative changes in I_TRPV1_ by gintonin at −100 mV. **** *p* < 0.0001 vs. control.

**Figure 7:**
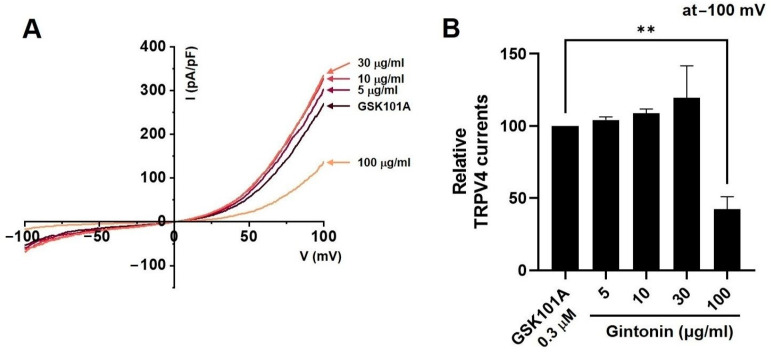
Effects of gintonin on overexpressed transient receptor potential vanilloid (TRPV)4 channel currents in HEK293T cells. (**A**) Representative I-V curve demonstrating the effects of various gintonin concentrations on overexpressed TRPV4 currents (I_TRPV4_). (**B**) Statistical analysis of relative changes in I_TRPV4_ by gintonin at −100 mV. ** *p* < 0.01 vs. control.

**Figure 8:**
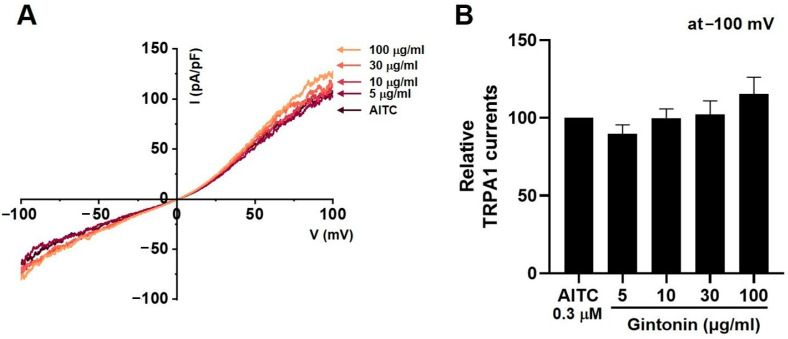
Effects of gintonin on overexpressed transient receptor potential cation channel subfamily A member 1 (TRPA1) channel currents in HEK293T cells. (**A**) Representative I-V curve displaying the effects of various gintonin concentrations on overexpressed TRPA1 currents (I_TRPA1_). (**B**) Statistical analysis of relative changes in I_TRPA1_ by gintonin at −100 mV.

Capsaicin (CAP) was used as an agonist for TRPV1 current (I_TRPV1_) [20]. After the application of 1 µM CAP to the bath, I_TRPV1_ was evoked. The application of 30 μg/mL (*n* = 14) gintonin inhibited the I_TRPV1_ increase (*p* < 0.0001), but the application of 5 (*n* = 12), 10 (*n* = 11), and 100 μg/mL (*n* = 12) gintonin had no effect on I_TRPV1_ (Figure 6A). Figure 6B displays the relative TRPV1 currents in response to gintonin at −100 mV. GSK101A was used as an agonist for TRPV4 current (I_TRPV4_) [21]. After the application of 0.3 µM GSK101A to the bath, I_TRPV4_ was evoked. The application of 5 (*n* = 11), 10 (*n* = 15), 30 μg/mL (*n* = 12) gintonin did not affect I_TRPV4_, but this current was inhibited by 100 μg/mL gintonin (Figure 7A). Figure 7B displays the relative I_TRPV4_ at −100 mV in the presence of gintonin. In addition, allyl isothiocyanate (AITC) was used as an agonist for TRPA1 current (I_TRPA1_) [22]. After application of 30 µM AITC, I_TRPA1_ was evoked, but was not affected by 5 (*n* = 11), 10 (*n* = 14), 30 (*n* = 13), or 100 μg/mL (*n* = 11) gintonin (Figure 8A). Figure 8B displays the relative I_TRPA1_ at −100 mV after the application of gintonin. These results suggest that TRPV1 and TRPV4 may be involved in the regulation of IBS-induced visceral hypersensitivity.

### 2.7. Effects of Gintonin on NaV1.5 and NaV1.7 Channel Currents

**Figure 9:**
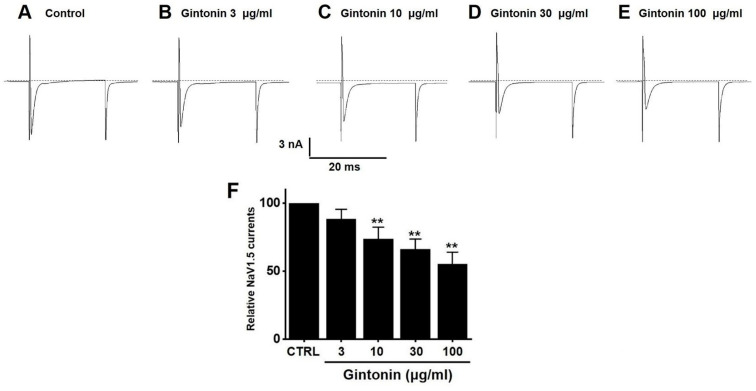
Effects of gintonin on overexpressed voltage-gated sodium (NaV1.5) currents in HEK293T cells. Representative plot displaying the effects of gintonin at different concentrations, (**A**) control, (**B**) 3, (**C**) 10, (**D**) 30, and (**E**) 100 μg/mL, on overexpressed NaV1.5 currents. (**F**) Statistical analysis of relative changes of NaV1.5 currents by gintonin. ** *p* < 0.01 vs. control.

**Figure 10:**
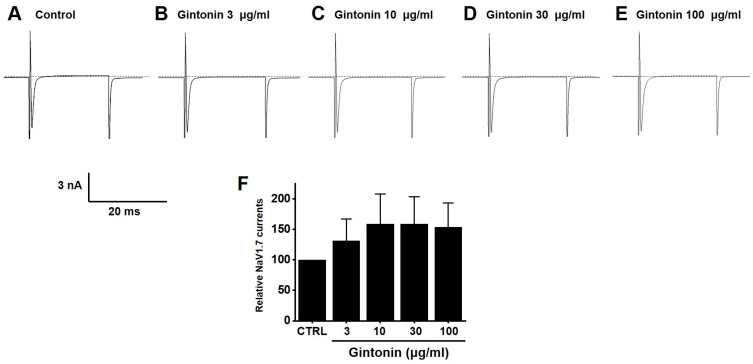
Effects of gintonin on overexpressed voltage-gated sodium (NaV1.7) currents in HEK293T cells. Representative plot demonstrating the effects of gintonin at different concentrations, (**A**) control, (**B**) 3, (**C**) 10, (**D**) 30, and (**E**) 100 μg/mL, on overexpressed NaV1.7 currents. (**F**) Statistical analysis of relative changes of NaV1.7 currents by gintonin.

Gintonin inhibited NaV1.5 currents. Currents were 88.32 ± 7.27% (*n* = 13) at 3 μg/mL, 73.73 ± 8.76% (*p* < 0.01; *n* = 11) at 10 μg/mL, 66.25 ± 7.47% (*p* < 0.01; *n* = 12) at 30 μg/mL, and 55.23 ± 8.96% (*p* < 0.01; *n* = 13) at 100 μg/mL compared to the baseline (Figure 9). In contrast, gintonin showed a slight increase in NaV1.7 currents, although it was not statistically significant at the concentrations tested (3 (*n* = 14), 10 (*n* = 10), 30 (*n* = 12), and 100 μg/mL (*n* = 12)) (Figure 10). These results suggest that NaV1.5 currents may be involved in the regulation of IBS-induced GI function.

## 3. Discussion

IBS is a dysfunctional GI disease with a high prevalence that is characterized by abdominal pain and discomfort, fecal abnormalities, and bloating. Pathogenesis is complex, and the exact molecular pathophysiology is not well understood [1,2,3]. Altered visceral sensitivity, intestinal motility, secretion dysfunctions, psychiatric comorbidities, GI immune abnormalities, intestinal bacterial imbalances, and mucosal dysfunctions are all associated with IBS [4,7]. The initial crucial step in IBS treatment is to psychologically reassure the patient. The second is to correct local factors that stimulate movement and sensation in the GI tract. The third is to note the prevalent symptoms and treat the individual accordingly [23,24]. Dietary habits and lifestyle factors can initiate IBS symptoms. Furthermore, proper diet and nutrition are essential in order to prevent malnutrition and control symptoms in patients with IBS [24,25]. Regular meals, sufficient water intake, and intake of suitable food types are imperative [24,25]. In addition, treatment with antidiarrheals, antispasmodics, and/or antidepressants is needed to relieve the IBS symptoms [2,23]. In other words, tailoring the diet and drug treatment to address each patient’s main symptoms is necessary, and successful treatment will improve the patient’s quality of life [1,2,3,4,7,23].

Gintonin is an LPA receptor ligand that activates G protein-coupled receptors that are key to the biological effects of gintonin [11]. LPA is present in several organs, including the GI tract [26]. The efficacy of gintonin has only been studied in the GI tract so far, where gintonin depolarized pacemaker potentials in small intestinal ICC through LPA1 and LPA3 receptor signaling pathways and the lipid component of gintonin mediated its absorption into the intestine by diffusion or active transport [14]. In addition, gintonin has a protective effect against gastric ulcers [15]. Although gintonin has various functions in the GI tract, studies on IBS have not yet been conducted. In the present study, we examined the efficacy of gintonin in alleviating the symptoms of IBS. Gintonin restored colon length and weight and decreased stool score in a murine model of zymosan-induced IBS (Figure 1). Throughout the experiment, food intake was constant and gintonin prevented the weight loss induced by zymosan treatment (Figure 2). The thickening of colonic mucosa, increase in TNF-α (Figure 3), and pain-related behaviors (Figure 4) were all reduced by gintonin. Moreover, gintonin altered the fecal microbiome: feces from the gintonin-treated mice had relatively high levels of *Lactobacillaceae* and *Lachnospiraceae* and low levels of *Bacteroidaceae* (Figure 5). The large intestine is rich in microbiota that are also distributed throughout the GI tract, the esophagus, and the stomach [27]. The gut microbiota is very closely related to our overall health status, and changes in the composition of the microbiota are associated with various GI and non-GI diseases [28,29]. The gut microbiota is altered in IBS, and the decrease in intraluminal bacterial diversity is involved in IBS pathophysiology [30]. The effectiveness of fecal microbiota transplantation to improve the dysbiosis observed in IBS is currently under investigation [31], and several studies have demonstrated that *Lactobacillaceae* supplementation can help relieve IBS symptoms [32]. In addition, the relative distribution of *Lachnospiraceae* is significantly reduced, and *Bacteroidaceae* is increased in IBS [33,34,35]. In this study, the composition of microorganisms in the mouse group with zymosan-induced IBS was different from those in the naïve non-IBS and the gintonin groups at the phylum level (Figure 5A). Gintonin also changed the fecal microbial composition at the family level: *Ruminococcaceae* did not change (Figure 5B) and *Lactobacillaceae* (Figure 5C) and *Lachnospiraceae* increased (Figure 5D), although this was not a significant change. However, *Bacteroidaceae* were reduced, although not significantly (Figure 5E). The part where the results differed significantly depending on the experimental state was the study of microbiota. It was quite difficult to keep the animal condition constant at all times, and it seems that the results were often different due to various stresses during drug treatment. In this study, there were some that did not have many differences in the results. In the future, we will conduct microbiota-related research more carefully. Therefore, gintonin appears to have the ability to normalize the distribution of gut microbiota, thus alleviating the symptoms of IBS. However, this experiment is a study confirming the efficacy in an IBS model with the type of diarrhea caused by zymosa as the main symptom. Therefore, it is thought that the efficacy of other types of IBS, such as constipation, should also be checked, and in the end, the efficacy should be confirmed in the human body. Also, only 100 and 250 mg/kg concentrations of gintonin were tested, and in the future, the concentration will be reduced or increased to about 50 or 500 mg/kg to confirm the efficacy.

Gintonin inhibited the TRPV1 (Figure 6) and TRPV4 (Figure 7) channels, but had no effect on TRPA1 (Figure 8). Additionally, gintonin inhibited NaV1.5 (Figure 9), but not NaV1.7 channels (Figure 10). The present study confirmed that gintonin might be effective in the treatment of IBS due to inhibition of TRPV1, TRPV4, and NaV1.5 channels. Various receptors in the GI tract are becoming new targets for IBS treatment [36]. In particular, TRP and NaV channels are the subject of much research, being widely distributed throughout the GI tract, with important roles in maintaining physiological function [36,37,38]. Studies on the roles of TRP and NaV channels in various diseases are ongoing, and new clinical applications are particularly focused on the role of these channels in pain suppression in IBS [36,37,38,39]. Visceral hypersensitivity in IBS is believed to cause visceral pain. TRPV1, TRPV4, TRPA1, NaV1.5, and NaV1.7 are ion channels that affect the visceral hypersensitivity of IBS [36,40]. TRPV1 is a well-known capsaicin receptor with calcium permeability [41,42] that is widely prevalent in the GI tract [43]. The expression of TRPV1 increases with age in the stomach and small intestine and is significantly decreased in patients with ulcerative colitis [44]. TRPV4 is a non-selective cation channel activated by temperature, acidic pH, and mechanical and chemical stimulations [42,45]. TRPV4 is present in the GI tract [46]. Furthermore, TRPV4 is expressed in human colon epithelial cells and the Caco-2 human colon cancer cell line, and is increased in inflammatory bowel disease [47,48]. TRPA1 is activated by chemical stimuli such as menthol, cinnamaldehyde, AITC, and allicin, as well as physical stimuli, e.g., temperatures < 17 °C [49]. TRPA1 acts as a chemical sensor of the intestinal environment in the GI tract and regulates GI functions such as gastric contraction, abdominal pain, GI motility, and secretion [50]. Interestingly, in rodents, TRPA1 is predominantly colocalized with TRPV1, indicating an interaction between these two ion channels. Additionally, TRPA1 also plays a role in intestinal immunomodulation [51,52]. NaV channels are expressed in the GI smooth muscle of various animals [44,53,54]. NaV1.5 channels are present in the ICC and smooth muscle of the small and large intestines [55,56]. Interestingly, patients with arrhythmias due to mutations in NaV1.5 channels often exhibit significant GI symptoms, and those with mutations in NaV1.5 channels associated with arrhythmias show symptoms of IBS [37,57]. This suggests that NaV1.5 channels not only are involved in cardiac function, but also play an important role in GI function. Particularly, patients with NaV1.5 channel mutations who show symptoms related to GI motility imply that these channels may be crucial in regulating gut motility [37,57]. These patients may experience symptoms such as constipation, abdominal pain, and bloating, which could be due to the role of NaV1.5 channels in regulating the electrical activity of the GI tract. Such findings indicate that NaV1.5 channels could be a potential therapeutic target for GI disorders. If drugs that modulate NaV1.5 channels are developed, they could be used to treat not only arrhythmias, but also GI diseases. In addition, NaV1.7 is an ion channel associated with several pain syndromes [39]. Treatment targeting NaV1.7 channels is emerging as a significant clinical objective, with promising outcomes linked to pain management [39]. Recent studies have suggested that NaV1.7 plays a role in visceral hypersensitivity of IBS, and, therefore, NaV1.7 inhibitors could be a viable treatment option to treat chronic visceral pain in IBS [39]. In the present study, gintonin inhibited the TRPV1 (Figure 6) and TRPV4 (Figure 7) and had no effect on the TRPA1 (Figure 8). In addition, gintonin inhibited the NaV1.5 (Figure 9) and had no effects on the NaV1.7 (Figure 10). suggesting that the reduction in IBS due to gintonin can be attributed to TRPV1, TRPV4, and NaV1.5. Comparing the effects of by Banhasasim-tang, a representative herbal medicine used in the GI tract [7], it can be seen that the changes related to IBS, such as colon length and weight, are similar, but the related ion channel mechanisms are different. TRPA1, NaV1.5, and NaV1.7 ion channels are involved in Banhasasim-tang [7], but TRPV1, TRPV4, and NaV1.5 are involved in gintonin. Therefore, various ion channels are involved in IBS, and it is thought that a combination of these herbal medicines can have synergistic effects. Therefore, treatment of IBS through a combination of prescriptions can achieve favorable results. From this perspective, gintonin may be a safe and effective drug to treat IBS through a single prescription or in combination with other drugs.

The effect of gintonin on NaV may also be observed in the enteric nerves. It is likely that gintonin increases the NaV1.7 current and suppresses the NaV1.5 current in the enteric nerves, leading to a net effect where the suppression of NaV1.5 is compensated for by the increase in NaV1.7. In addition, it should be considered that the response in NaV results can vary depending on the species. First of all, when comparing the alignment between mouse and human NaV1.5, there does not seem to be a significant difference in the NaV1.5 that showed an inhibitory response in this study. Additionally, according to Reinhard et al. [58], the electrophysiological properties between mouse and human NaV1.5 are quite similar, with no significant differences except for the reversal potential, the slope of the inactivation curve, and the degree of recovery from inactivation. However, despite these similarities, we think that the effects could still vary depending on the drugs. The effects of NaV channel blockers can be compared across multiple species to derive correlations between humans and mice. In this study, we investigated the efficacy of gintonin using human NaV channels. In the future, we will obtain mouse NaV channels to verify the efficacy of gintonin and identify any differences between human and mouse NaV channels.

The biologically active components of gintonin are LPAs. Gintonin LPAs exert their effects by binding to the LPA 1/3 receptor subtypes on the cell membrane. In a previous in vivo study on the gintonin-induced opening of the blood–brain barrier (BBB), it was observed that when 40 mg/kg of gintonin was administered intravenously to rats, plasma LPAs (LPA C18:2, LPA C16:0, and LPA C18:1) peaked within a short period of about 2 min. These elevated blood gintonin LPAs are expected to exert biological effects [59]. We have not previously studied the specific free plasma Cmax levels achieved after 250 mg/kg gintonin. However, although there are differences, such as the animal model changing from rats to mice, the concentration increasing approximately sixfold from 40 mg/kg to 250 mg/kg, and the administration route changing from IV to oral, the levels of blood gintonin LPAs are still expected to increase within a short time. Moving forward, we plan to confirm changes in plasma LPA levels after oral administration of 250 mg/kg to mice. Additionally, one might wonder how ginseng-enriched fraction (GEF), a novel ginseng material rich in gintonin, can be effective for IBS when it is only included at 1.3%. Our results do not ignore or exclude the potential biological effects of the remaining 98% of other components present in ginseng. Instead, our study emphasizes that, even though the proportion of GEF is small, it is highly concentrated with LPAs, which are LPA receptor ligands (0.2%), and also contains and is enriched with other phospholipids and fatty acids that can serve as LPA precursors [11]. This distinguishes it from the remaining 98% of ginseng components.

Further investigation into the precise mechanisms by which gintonin regulates ion channels and alleviates IBS symptoms is crucial. Understanding the molecular pathways involved will enhance the development of targeted therapies. Also, determining the optimal dosage and administration route of gintonin for maximum efficacy and minimal side effects is essential. Conducting long-term studies to evaluate the safety and efficacy of gintonin in treating IBS over extended periods is necessary. This will help in understanding any potential long-term effects or benefits. In addition, exploring the potential of gintonin in combination with other treatments for IBS could provide synergistic effects and improved outcomes for patients, and identifying biomarkers that predict response to gintonin treatment will help in personalizing therapy and improving treatment outcomes. By focusing on these areas, we can better understand the therapeutic potential of gintonin and accelerate its translation into clinical practice for the benefit of IBS patients.

Additionally, there are some limitations in this study, and we think that they should be considered in the future. The use of animal models, such as zymosan-induced inflammation in mice, may not fully replicate the complexity of human IBS. Differences in physiology, immune responses, and gut microbiota between animals and humans can limit the translatability of the findings. While zymosan-induced models mimic some aspects of IBS, they may not capture the full spectrum of symptoms experienced by IBS patients, such as psychological stress or chronicity. Also, although the study identifies potential ion channels (TRPV1, TRPV4, and NaV1.5) regulated by gintonin, the precise molecular pathways and interactions remain insufficiently explored. Further in-depth studies are needed to elucidate the detailed mechanisms of action. In addition, IBS is a heterogeneous disorder with various subtypes. The study does not address how gintonin affects different IBS subtypes, and future research should consider this variability to tailor treatments more effectively.

In summary, zymosan activates the immune response, leading to inflammation, which is characteristic of IBS. Gintonin may exert anti-inflammatory effects by inhibiting the release of pro-inflammatory cytokines (e.g., TNF-α). This anti-inflammatory action may help in mitigating intestinal inflammation and related symptoms. Gintonin is known to interact with LPA receptors, which are involved in various cellular functions, including cell proliferation, survival, and migration [11]. LPA signaling in the gut may influence smooth muscle contraction, secretion, and barrier function. By modulating LPA receptor activity, gintonin could contribute to the restoration of normal GI function. TRPV1 and TRPV4 channels are involved in the sensation of pain and thermal stimuli [36,40]. Gintonin’s ability to modulate these channels can reduce visceral hypersensitivity, a key symptom of IBS. By decreasing TRPV1 and TRPV4 activity, gintonin may alleviate abdominal pain and discomfort. NaV1.5 channels are present in the ICC and smooth muscle of the small and large intestines [55,56]. Interestingly, dysregulation of NaV1.5 has been linked to altered GI motility seen in IBS [37,57]. Gintonin’s regulatory effect on NaV1.5 could normalize motility patterns, thereby reducing symptoms like diarrhea or constipation.

## 4. Materials and Methods

### 4.1. Gintonin Preparation

Gintonin was extracted from *P. ginseng* as previously described [11]. One kilogram (4 years ginseng) was crushed and refluxed eight times with 70% ethanol for 8 h at 80 °C. The ethanol extract (350 g) was concentrated, diluted in cold distilled water, and stored at 4 °C for 24 h. After centrifugation of the extract, the precipitate was lyophilized. This GEF displayed a yield of 1.3%, and the chemical composition of GEF was identified previously [11].

### 4.2. Animal Experiments

Male C57/BL6 mice (Samtako Bio, Osan, Republic of Korea) weighing 20–25 g were used in the analysis. To induce colitis, 0.1 mL zymosan was injected directly through the anus once daily for 3 days. The mice were categorized into six distinct groups: (a) normal group (*n* = 18 mice), (b) PBS control group (*n* = 22), (c) AMT (Sigma-Aldrich, St. Louis, MO, USA) group (30 mg/kg, *n* = 21), (d) SSZ (Sigma-Aldrich, St. Louis, MO, USA) group (30 mg/kg, *n* = 19), (e) gintonin group (100 mg/kg, *n* = 21), and (f) gintonin group (250 mg/kg, *n* = 22). Gintonin was administered orally. The experimental design is summarized in Supplementary Material Figure S1. The animals were maintained under controlled conditions (21 ± 3 °C, relative humidity 52% ± 3%, lights on 6 a.m.–6 p.m.). Euthanasia of the mice was performed by decapitation after anesthesia with CO_2_. After that, the necessary tissues were collected and the experiment was conducted.

### 4.3. Change in the Colon by Zymosan

On day 4, the weight and length of the colon were measured after fecal removal. Colon weight was measured after defecation was removed, and colon length was measured from the anus to the cecum. Three researchers scored 0 for normal, 1 for moist, 2 for sticky, and 3 for diarrheal regarding stool condition.

### 4.4. Changes in Body Weight and Food Intake

Body weight was measured on days 1, 4, 9, and 12. Food was weighed at the same time each day, and total food intake was calculated for the 12 days.

### 4.5. Identifying Histological Characteristics

On day 12, tissue was obtained by biopsy from the colon, fixed with paraffin, cut to a thickness of 4 μm, and stained with hematoxylin and eosin (H&E). Moreover, a light microscope (Nikon, Tokyo, Japan) was used.

### 4.6. Enzyme-Linked Immunosorbent Assay (ELISA) Measurement

TNF-α was measured at 12 days in the homogenate of colon tissues using a commercial ELISA kit (BD Biosciences, San Diego, CA, USA).

### 4.7. Measuring of Pain-Related Behavioral Changes

Pain-related behaviors were assessed as previously described [60]. Two or more researchers observed the mice, and the total number of pain-related behaviors, such as licking the abdomen, pressing the abdomen to the floor, and stretching the entire body, was recorded over 10 min.

### 4.8. Microbiota Analysis

To obtain genomic DNA from stool samples, the QIAamp PowerFecal DNA Kit (QIAGEN, Hilden, Germany) was used. The bacterial 16S rRNA V4 region was amplified with unique 8 bp barcodes and sequenced on the Illumina iSeq 100 system [61]. The sequences were processed into the Quantitative Insights into Microbiological Ecology (QIIME) pipeline using the SILVA 128 database [62,63].

### 4.9. Electrophysiology

Human TRPV1, human TRPV4, human TRPA1, and human NaV plasmids were transiently transfected into HEK 293T cells with pEGFP to identify transfected cells. Data were obtained using an Axopatch 200B amplifier (Molecular Devices, San Jose, CA, USA) connected to a Digidata1440A (Molecular Devices, San Jose, CA, USA) and organized using pCLAMP 10.7 (Molecular Devices, San Jose, CA, USA). The external and internal solutions and the patch clamp techniques were described in a past study [7]. To determine whether gintonin regulates ion channel activity, we performed whole-cell electrophysiological recordings on overexpressed ion channels in HEK293T cells. To verify the current–voltage (I–V) relationship, a ramp pulse ranging from −100 mV to +100 mV was used for the TRP channel, while for the NaV channel, the voltage was held at −120 mV for 20 ms and then changed to 0 mV for 20 ms to measure the response.

### 4.10. Statistical Analysis

The results are presented as mean ± standard error of the mean (SEM), and Tukey’s multiple comparison test was used for the analysis of variance using GraphPad Prism (version 6). Statistical significance was set at *p* < 0.05.

## 5. Conclusions

In this study, gintonin restored colon length and weight, stool scores, and body weight in mice with zymosan-induced IBS. Inflammation-related TNF-α levels and pain-related behaviors returned to normal. In addition, the inhibition of TRPV1 and TRPV4 by gintonin may have contributed to the reduction in visceral hypersensitivity, while the inhibition of NaV1.5 by gintonin may have played a role in the regulation of GI function. Therefore, treatment of IBS through a combination of prescriptions can achieve favorable results. From this perspective, gintonin may be a safe and effective drug to treat IBS through a single prescription or in combination with other drugs.

## Data Availability

The original data are available upon reasonable request to the corresponding author.

## Supplementary Materials

The following supporting information can be downloaded at: https://www.mdpi.com/article/10.3390/ph17091170/s1, Figure S1: Experimental design.
