# Supplementary figures and images for: Study on the Therapeutic Effects and Mechanisms of Gintonin in Irritable Bowel Syndrome and Its Relationship with TRPV1, TRPV4, and NaV1.5
Source: Pharmaceuticals (Basel). 2024 Sep 4;17(9):1170. doi: 10.3390/ph17091170 (PMC11435028; doi:10.3390/ph17091170)

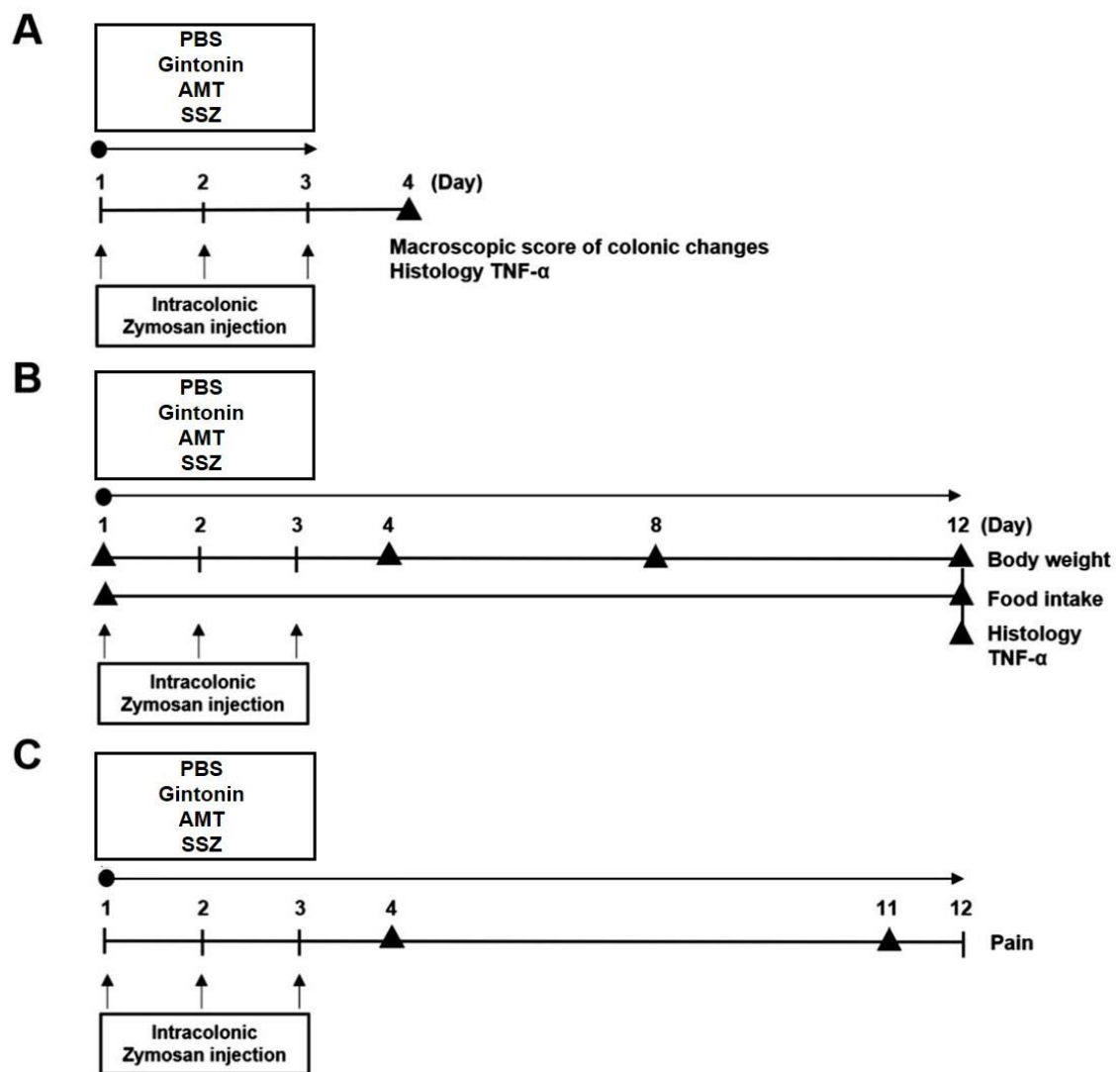

Supplementary Material Figure S1. Experimental design.

Supplement: Supplementary file 1 [file pharmaceuticals-17-01170-s001.zip › pharmaceuticals-3129493-supplementary.pdf]
